# A commentary on “Effect of parecoxib on postoperative delirium in patients with hyperlipidemia: a randomized, double-blind, single-center, superiority trial”

**DOI:** 10.1097/JS9.0000000000002674

**Published:** 2025-06-05

**Authors:** Junhui Zhou, Wei Zhong, Gaoyuan Xi

**Affiliations:** Department of Anesthesiology, Henan Provincial Chest Hospital (Chest Hospital of Zhengzhou University), Zhengzhou, Henan, People’s Republic of China.

*Dear Editor*,

We read with keen interest the randomized controlled trial (RCT) conducted by Daojun *et al*[1], recently published (February 2025) in the *International Journal of Surgery*. This RCT offers important insights into the effectiveness of preoperative parecoxib, a COX-2 inhibitor, for reducing postoperative delirium (POD) incidence in hyperlipidemic patients undergoing colorectal surgery. With a sample size of 452 and a 90% statistical power, the study reported a notable 12.39% absolute risk reduction and a substantial 47.4% relative risk reduction in POD. This reduction is mediated through prostaglandin endoperoxide synthase-2 (PTGS2) suppression and improved analgesia. The reliability of the study was further bolstered by the use of the 3-minute diagnostic interview for the Confusion Assessment Method (3D-CAM) scale, as well as comprehensive subgroup analyses. These analyses highlight benefits in patients with limited education, mild hyperlipidemia, or the absence of carotid stenosis.

Although the single-center design of this study enhanced internal validity through standardized protocols, it inherently limits generalizability. The homogeneous cohort, involving hyperlipidemic patients undergoing colorectal surgery, may not capture the variability seen in broader surgical populations or diverse ethnic groups. Furthermore, multicenter studies have shown that POD risk factors can vary significantly depending on surgical context and patient characteristics[2]. In addition, the effectiveness of parecoxib may also vary by surgical type[3]. For example, extended cardiopulmonary bypass in cardiac surgery may blunt pharmacological effects, while trauma severity can influence outcomes in orthopedic cases. Patients with head and neck cancer often require tailored nutritional strategies, which can influence POD risk. Furthermore, population-specific factors complicate extrapolation: patients with lower educational attainment may benefit more due to reduced cognitive reserve, whereas those with vascular comorbidities or severe hyperlipidemia may respond less due to impaired cerebral perfusion or metabolic dysregulation. Thus, the findings are most applicable to elderly colorectal surgery patients with mild hyperlipidemia and no major vascular complications and should be validated in broader, multicenter trials with biomarker-driven stratification.

Another concern is the potential underdetection of transient or nocturnal delirium. POD assessments were conducted once daily in the evening between 4:00 PM and 8:00 PM, possibly missing brief or nighttime episodes of delirium. In our view, it would be beneficial to incorporate continuous monitoring tools, such as the validated Confusion Assessment Method for the Intensive Care Unit, which allows hourly evaluations[4]. Furthermore, while systemic PTGS2 and leukocyte counts were evaluated, such blood-based markers may not fully represent the neuroinflammatory processes integral to POD pathogenesis. The lack of central nervous system (CNS)-specific markers limits mechanistic insights. It will be useful to assess CNS neuroinflammatory markers such as interleukin 6 or glial fibrillary acidic protein to gain better mechanistic insights into CNS disorders such as POD. These gaps highlight the need for better study designs to capture the complexity of delirium mechanisms and their clinical manifestations.

Moreover, the 3-month follow-up duration limits the ability to draw conclusions regarding long-term cognitive impacts of parecoxib, specifically its potential to alleviate postoperative neurocognitive disorder. Therefore, longitudinal studies and cost-effectiveness analyses (e.g. hospital stay duration, rehabilitation expenses, and overall healthcare utilization) are necessary to support its widespread clinical application. Mechanistic studies that prioritize single-cell RNA sequencing to map the influence of parecoxib on neuro-immune crosstalk, complemented by the use of appropriate animal models, may further elucidate its effects on neuroimmune pathways and blood-brain barrier integrity. Although AI was not used in this study, its role in clinical research is growing. The recently published TITAN guideline provides a framework to ensure reproducibility and ethical standards in AI-supported research and will be valuable for future studies, especially in shaping scientific workflows[5].

In conclusion, the RCT conducted by Daojun *et al*[1] provides compelling evidence for the use of preoperative parecoxib in reducing POD incidence in patients with hyperlipidemia. While its findings are promising, broader multicenter trials with diverse populations, extended follow-up, and CNS-specific biomarker integration are essential to confirm and refine targeted POD prevention strategies.

## Data Availability

As this manuscript is commentary, no original datasets were generated. All data referenced in this study are publicly available from cited sources.
